# Proof of the Periodical Ripening and Separation of the Ova of Mammiferæ and Man, Independent of Coitus, Being the First Condition of Their Propogation

**Published:** 1845-03

**Authors:** 


"*Proof of the Periodical Ripening and Separation of the Ova of Mammalia and Man, independent of Coitus, being the first condition of their Propagation.* By Th. L. W. Bischoff, M. D., P. D., &c., Giessen, 1844."

The review begins with a few introductory remarks as follows:

"The patience and industry with which the comparative anatomist, aided by the microscope, has pursued the study of the intricate phenomena of animal life, through every stage and form of existence, and in the most minute structures, have been richly rewarded by the attainment of results as important, as in many instances they were unexpected. In no branch of physiology is this remark more true than in that which relates to generation; a subject of surpassing interest, and, within a very recent period only, shrouded in the darkest mystery and uncertainty. It is true, that much entirely impenetrable by our present means of investigation, remains still to be learned, but yet many steps in advance have been made, which may encourage to farther labor and investigation. The discovery, that the Graafian vesicle, instead of being a glandular body, destined to secrete a seminal fluid, actually contained an ovum, differing in the mammalia in no essential respect from those of the inferior orders of animated beings, was of the utmost moment, by removing one of those distinctions which it was supposed existed between these orders. Still it was alleged that sexual intercourse was requisite to cause the formation of the Graafian vesicle. Investigation proved that they were found in virgins and even in infants. It was next asserted, that if sexual intercourse was not requisite to their formation, it was absolutely essential to the maturation and separation of the ova; driven again from this position, by the fact that in virgins, *corpora lutea*, which are the remains of ruptured vesicles, have been found, as a last resort, the physiologist was compelled either to call to his aid the influence of a high state of sexual excitement, or to deny that true *corpora lutea* are formed in virgins, pointing out, at the same time, the means by which the true might be distinguished from the false.

"The labors of distinguished men have, at length, solved all these difficulties, by showing conclusively, that at the period of heat in the mammalia generally, and at each menstrual period in woman, an ovum, which has been gradually developing itself in a Graafian vesicle of the ovary, becomes mature, bursts from the vesicle and passes tnto the oviduct or Fallopian tube, whether sexual intercourse takes place or not, (indeed, that this whole phenomenon is entirely independent of cöitus,) and that after having passed into the oviduct, it may be impregnated if connection takes place, and the seminal fluid reaches it, or it may be lost.

"But we are anticipating our subject, and must proceed to direct attention to the works above named, which are devoted to the relation of the experiments and conclusions of the authors on the subject of generation. As however, the work of M. Raciborski embraces a wider range, taking up the subject of menstruation in a hygienic and medical point of view also, it shall first be presented to the reader, the discussion of the theory of the spontaneous periodical discharge of ova being deferred, though its truth must be assumed, until it regularly comes up in the course of this work, when it shall be considered in connection with the same doctrine as developed in the works of M. Pouchet, and of M. Bischoff, which are more exclusively occupied with the proofs of its truth.

"An abstract of some of the conclusions to which M. Raciborski had been conducted, respecting the causes which influence the period of puberty, with one of the valuable tables upon which these conclusions were based, was laid before the readers of this Journal, in the April number of 1844, having been extracted from the valuable anatomical report of Mr. Paget. A brief recapitulation of these will suffice for our purpose and will serve as an introduction to much other matter relative to menstruation, which we would willingly give *in extenso*, so well is it brought together."

This recapitulation, and that which follows, being the review of those portions of M. Raciborski's work relating to "Marriage in a medical and social point of view;" "The hygiene of young girls, at the approach of puberty;" "the different affections which may retard or interfere with the development of the Graafian vesicles, and thus prevent menstruation;" we are obliged to omit, though replete with interest and practical importance, until some future occasion. The following remarks, however, bear such relations to other parts which are to be presented, that we extract them:

"The revolution of the economy which is observed at the age of puberty, is, as is well known, a very marked one even to the most superficial observer. M. Raciborski considers, that 'the most unequivocal sign of procreative maturity, nay, even more certain than the menstrual evacuation itself, which, from its resemblance to other hæmorrhages, may sometimes lead to error, is the development of the mammary glands, of the pelvis, and of the external sexual organs,' (p. 97,) especially the covering of the pubes with hair, for it is well ascertained, he says, that when these changes occur, the Graafian vesicles have arrived at a degree of development, at which 'they only await the command to burst and cause the first laying, (*ponte*,) which almost always coincides with the first eruption of the menses;' though such a *ponte* may, and probably does occur, he says, before any hæmorrhage is observed, where girls experience periodically, for several months before their menses appear, colics and pains in the abdomen and loins, &c., p. 98.

"In a note to his description of the ovaries, contained in this chapter, M. R. says, that to dissect an ovary properly, it should be steeped in alcohol or boiling water, to harden the albuminous liquid in the vesicles. This done, three incisions should be made through it, parallel to its long diameter, one along the median line, and one on each side; the two lateral incisions being close to the external envelop, present a large number of round depressions, filled, most of them, with a cheesy matter, white, or sometimes a little red—if a little blood is mixed with the albumen—of different sizes, rather more numerous on the anterior than the posterior surface of the ovary, which are, in fact, the Graafian follicles divided; whereas, upon the median incision, that which is usually alone made, but few will be found, and chiefly on the upper edge. By this mode of proceeding, some thirty or forty follicles will be discovered in each ovary of a young woman, at the period of puberty; these follicles being disseminated around the proper substance of the ovary, which forms a central nucleus."

We proceed with our extracts from the review:

"We are thus brought to the consideration of the facts upon which this new doctrine of generation is based; a doctrine which, by a coincidence not rare in the annals of science, is promulgated almost at the same moment by a number of distinguished men, whose occupations and pursuits have brought them by different trains of reasoning to the discovery, probably, in several instances, without a knowledge of each others labors. It is true, that the important truth had been foreshadowed, and even indistinctly perceived by recent investigators, as M. M. Négrier, Paterson, R. Lee, Jones, &c., who were, however, too much wedded to old opinions to yield themselves immediately to the new light which was breaking in upon them; and it remained for M. Pouchet, the Professor of Zoology at the Museum of Natural History of Rouen, to give the first distinct and positive enunciation of the doctrine. In his work, whose title is given above, published in 1842, a work stamped with the impress of profound thought, clear perception, and thorough knowledge of the subject on which he was writing, M. Pouchet has developed, having taught it to his class since' 1835, "the positive theory of fecundation of the mammiferæ,' with all the enthusiasm and energy of conviction. He has even anticipated and answered almost every objection; and although some few errors may be discovered, they must be attributed rather to the fact that his conclusions are based upon a learned scrutiny of the records of science, than upon actual experiments of his own relative to this point.

"His conclusions are thus summed up in ten fundamental and three accessory physiological laws, the latter of which, though he considers them proved, may admit of argument. In a subsequent portion of our notice the general accuracy of these will be shown, and some of their probable fallacies pointed out.

"'1st Fundamental physiological law.—The human species forms no exception; the phenomena of its generation follow laws analogous to those which are observed among the different animals, and they are even perfectly identical with the acts which take place among those which occupy the head of the zoological series.

"'2d law.—Generation in all animals takes place by means of eggs, some inferior beings alone forming an exception.

"'3d law.—In the whole animal series the ovules pre-exist fecundation.

"'4th law.—Physical obstacles prevent, in the mammiferæ, the possibility of the seminal fluid being placed in contact with the ovules while yet contained within the Graafian vesicles.

"'5th law.—In the whole animal series, without the possibility of question, the ovary emits its ovules independently of fecundation.

"'6th law.—In all the animals the ovules are emitted at periods fixed, and in relation with the increased periodical excitement of the genital organs.

" '7th law.—In the mammiferæ fecundation never occurs unless the emission of the ovules coincides with the presence of the seminal fluid.

" '8th law.—The emission of the catamenial discharge of women corresponds with the phenomena of excitement which manifest themselves at the period of the *amours* of the different beings of the zoological series, and especially of the females of the mammiferæ.

" '9th law.—Fecundation offers a constant relation with the emission of the menses; thus, in the human species, it is easy to determine accurately the intermenstrual period at which conception is physically impossible, and that at which it may be probable.

" '10th law.—Assuredly there exist no ovarian pregnancies, properly so called.

" '1st Accessory physiological law.—Fecundation in the mammiferæ, is normally effected in the uterus.

" '2d law.—Abdominal and tubar pregnancies do not indicate that fecundation is effected normally *in* the ovary, and that it is this which causes the emission of the ovules.

" '3d law.—Normally, the Fallopian tubes only contract from within outwards to transport the ovules.'—p. 11, 12.

"Important as was the work thus performed, it was requisite, before full credence could be reposed in tnis new doctrine, that it should be sustained and established by direct experiment: this task MM. Raciborski and Bischoff both claim, each for himself, to have performed; the former, it is true, with a full knowledge of the writings of M. Pouchet; the latter, as is evident from his work, without any such knowledge to stimulate him in the prosecution of his undertaking. M. Duvernoy, also, asserts his claims to have discovered this relation between the Graafian vesicle and menstruation; and he unquestionably did, in the autumn of 1842, develop this theory briefly before the scientific Congress, which assembled at Strasburg that year, founding his opinions on analogical proofs.

"In support of their respective claims, the works whose titles have been placed at the head of this notice, have since appeared. Without pretending to decide positively respecting them, we cannot but think that the labors of M. Bischoff are best entitled to the merit of having experimentally proved the new doctrine, which he appears to us to have done in the most unquestionable manner. M. Raciborski, however, goes farther into the matter, not confining himself to the mere determniation of the law, but examining and illustrating some of its most important bearings, and applying it more strictly to women, upon whom his own observations appear chiefly to have been made.

"In the remarks which are to follow, we will take as our guide the truly experimental work of M. Bischoff. We have already observed that it was long supposed that the laws of generation in the mammalia and man differed essentially from those which were known to govern the other classes of animals. Though it was well ascertained that in these last the development, maturation and separation of the ova occur independently of the male, the contact of the male semen, however, being necessary for their final development—in the first it was supposed that the congress of the sexes was essential to give origin to the product which the female contributed for the future being. Recent discoveries, by disclosing the existence in the mammalia of ova, which, from their minuteness, had escaped detection by earlier observers, dissipated in part the supposed distinction, although the belief still remained that, notwithstanding the demonstrated existence of *corpora lutea* in the ovaries of virgins, the act of cöitus was requisite for the maturation and separation of the ova. The contact of the male semen was essential to fit them to be discharged.

"Fully impressed with the idea that the exit of the ova from the ovaria was the consequence of cöitus, or occurred during or soon after that act, M. Bischoff instituted a series of experiments for the purpose of ascertaining at what precise period the separation took place. He established, indeed, satisfactorily to himself, though M. Pouchet denies the possibility of such an occurrence normally, that in the dog, the semen penetrates in from twenty to twenty-four hours, and, in the rabbit, in from nine to ten hours, from the uterus, into which it is ejaculated during cöitus, through the oviduct to the ovaries. But he was, at the same time, convinced of his error in supposing that the separation of the ova was determined by copulation, though calculations, founded upon the above results, were approximatively accurate in determining the period of separation; for, as a general rule, none of these animals will allow themselves to be covered until the moment of separation has arrived, although the symptoms of heat may have continued for several days.

"The following fact was among those which first opened his eyes. He took a young, stout, healthy bitch, which had never been pregnant, and carefully watched her. About the beginning of June, 1843, symptoms of heat were apparent, the dogs following her, and there being a bloody discharge from the vagina. She would not receive a dog, however, until the 11th of June, when she was lined, and immediately afterwards M. B. removed, from the living animal, the left horn of the uterus, with the oviduct and ovary of the same side. Upon an examination, with the microscope, which was immediately made, living and moving spermatozoa were found in the upper extremity of this horn, though none could be found in the oviduct: but, to his astonishment, he discovered that five Graafian vesicles in the ovary had already burst, and *corpora lutea* were already distinctly formed; upon further examination, the five ova were fouud in the oviduct, having already advanced *two* inches, and resembling exactly *ovary-eggs*. The next morning, about twenty hours after copulation, at which time previous experiments had taught him the semen might reach the ovary, the bitch was killed; and, although spermatozoa were observed still alive in the right horn, they had merely penetrated into the end of the oviduct, about two and a half lines. Five ova were observed at about the middle of this canal, and several lines apart, resembling, generally, those found on the left side, but not as yet surrounded by spermatozoa; while, on the ovary corresponding, there were five fresh *corpora lutea* more advanced than on the other side, but still presenting the central opening.

"This experiment evidently proved that the separation of the ova had occurred previously to the copulation, for, as M. Bischoff remarks, copulation is now known never to have this effect, as he has found since, after several complete copulations of other bitches, that the Graafian vesicles were still entire, and besides, it is altogether improbable, that in a quarter of an hour, the ova could have advanced two inches in the oviduct, when they ordinarily require eight days to pass through its whole length of from two to three inches.

"By a repetition of the experiments of Nuck, Haighton, Cruikshank, Grassmayer, Blundell, &c., which consisted in interrupting, by ligature or otherwise, the communication between the uterus and ovaries through the oviduct—experiments which, in their hands, were productive only of negative results, showing that fecundation was impossible under such circumstances, although *corpora lutea* were formed—M. Bischoff shows, that though the advance of the male semen through the oviduct to the ova is prevented, the Graafian vesicles still swell and burst, *corpora lutea* are formed, the ova pass out, arrive in the oviduct, and, not being vivified, shrink and perish, and that 'hence, notwithstanding the intervention of copulation, the independent course of the development of the ova is proved.'—p. 18."

Here follow two experimental proofs, the one "proves most conclusively the maturation and separation of the ovum in a young virgin animal, consequently entirely independent of copulation;" the other "by a double experiment upon the same animal, proves as satisfactorily as possible, the ripening and separation of ova during heat and without the aid of the congress of the sexes."

M. Bischoff lays down the following law, "which it will be seen, brings the generation of the mammalia under the same general rule which obtains in that of all other classes of animals."

"'Both in mammalia and man, also, the self-forming ova in the ovaries of the female experience a periodical ripening, entirely independent of the influence of the male semen. At this period, which, in animals, is called heat, and in women, usually, menstruation, these matured ova separate themselves from the ovary and are pushed out. At this time, alone in animals, and especially in women, are sexual desires also manifested. If cöitus takes place, the fructification of the ovum follows, in consequence of the material action of the male semen upon it. If cöitus does not take place, the ovum does not the less separate from the ovary, pass into the oviduct, but there perishes. The relations of time, however, as it appears, may vary in different animals within different yet fixed limits. The semen may have sufficient time to reach the ovary before the ovum has separated. The ovum may also have already passed out, and the semen have first encountered it in the oviduct; the action of the semen upon the ovum must still, however, always take place, if the ovum is to be developed, which development is commenced while it is still in the oviduct. But at this period of the periodical ripening of the ova, alone, can cöitus be followed by fecundation.' "—pp. 4, 5.

The Review proceeds then with the proofs of the applicability of this law to woman. Our limits oblige us to leave the subject here, with the intention of continuing our extracts at another time.

